# Dietary supplementation with a high dose of daidzein enhances the antioxidant capacity in swine muscle but experts pro-oxidant function in liver and fat tissues

**DOI:** 10.1186/s40104-016-0102-z

**Published:** 2016-08-02

**Authors:** Wei Chen, Xianyong Ma, Yingcai Lin, Yunxia Xiong, Chuntian Zheng, Youjun Hu, Deqian Yu, Zongyong Jiang

**Affiliations:** 1 Institute of Animal Science, Guangdong Academy of Agricultural Sciences, Guangzhou, 510640, People’s Republic of China; 2 The Key Laboratory of Animal Nutrition and Feed Science (South China) of Ministry of Agriculture, Guangzhou, People’s Republic of China; 3 State Key Laboratory of Livestock and Poultry Breeding, Guangzhou, People’s Republic of China; 4 Guangdong Public Laboratory of Animal Breeding and Nutrition, Guangzhou, People’s Republic of China; 5 Guangdong Key Laboratory of Animal Breeding and Nutrition, Guangzhou, People’s Republic of China

**Keywords:** Anti-/pro-oxidant enzyme, Daidzein, Fat, Liver, Muscle, Pigs

## Abstract

**Background:**

Although isoflavones are natural dietary antioxidants, they may have toxicological effects. This study aimed to evaluate the redox system in tissues of finishing pigs by supplementation with high dose of daidzein (640 mg/kg).

**Results:**

The supplementation of high dose of daidzein for 64 d increased the activity of superoxide dismutase and total antioxidant capacity in longissimus muscle but down-regulated the expression of reactive oxygen species (ROS)-producing enzyme NADPH oxidase-2 and cyclooxygenase-2. In contrast, high-level supplementation with daidzein exerted pro-oxidant changes in back fat, abdominal fat, liver, and plasma, as reflected by increased contents of malondialdehyde, a lipid peroxidation product, in these tissues. Furthermore, daidzein supplementation resulted in higher expression of ROS-producing enzymes, including NADPH oxidase-1 and cyclooxygenase-1 in liver, 5-lipoxygenase (5-LOX) in backfat and NADPH oxidase-2 both in abdominal fat and backfat. The supplementation of daidzein did not affect meat quality parameters in longissimus muscle, including marbling score, eye muscle areas, intramuscular fat, shear force, drip loss, pH and meat color.

**Conclusions:**

This experiment suggests that dietary supplementation of finishing pigs with daidzein at a high dose level improves redox status in muscle but exerts pro-oxidant effect in liver and fat tissues.

## Background

Soy isoflavones, mainly composed of daidzein, genistein and glycitein, are known from in vitro studies to be active scavengers of hydrogen peroxide, hence acting as potential antioxidants. Because of this property [1, 2], isoflavones are considered to be natural dietary antioxidants with interesting benefits to health [3]; there is also potential for their use in animal production to improve growth performance [4]. There is, however, some controversy about the beneficial antioxidant effects of soy isoflavones because, for example, genistein and its methylated derivative, biochanin A, can mobilize nuclear copper in human lymphocytes, leading to degradation of cellular DNA [5]. There is increasing evidence for both antioxidant and pro-oxidant activities of isoflavones, depending upon the specific experimental conditions [6, 7]. These studies suggest a pro-oxidant potential of high concentrations of isoflavones.

Because of this pro-oxidant potential, one consideration for their application in practical animal production is whether dietary supplementation at high doses of isoflavones might change the redox system in muscle, thereby possibly affecting meat quality. Oxidative stress has been shown to reduce collagen solubility [8], possibly affecting toughness of meat. Direct evidence of negative consequences of high doses of isoflavones is still scarce in practical animal production. Accordingly, the objective of present study was to test the effects of dietary supplementation of high dose of the isoflavone daidzein on redox system in skeletal muscle, liver, and back fat and meat quality. The level of supplementation used here, 640 mg/kg feed, is 15 times higher than that providing optimal antioxidant function [4].

## Methods

The experimental protocol used in this study, including animal management, housing, and slaughter procedures, was approved by the Animal Care and Use Committee of Guangdong Academy of Agricultural Sciences.

### Animal and housing

A total of 48 hybrid finishing pigs (Duroc × Landrace × Large White, 24 gilts and 24 barrows) averaging 57 kg BW were obtained from a single source (a commercial swine farm in South China) and transported to the trial farm. Pigs of each sex were initially blocked into 2 weight groups then randomly assigned to either treated or control groups, each consisting of 6 pens (2.5 m × 2.5 m), of 4 pigs (2 barrows and 2 gilts) of comparable total starting BW. Pigs in the control group were fed a basal diet that met the NRC [9] recommendation for finishing phase (Table 1). The treated pigs received the basal diet supplemented with 640 mg/kg daidzein, provided by Guangdong Newland Feed Science & Technology Co., Ltd.

**Table 1:** Composition of the basal diet fed to finishing pigs (as-fed basis)

Item	Content
Ingredient	
Corn	68.7
Soybean	22.1
Wheat middlings	6.0
Monocalcium phosphate	0.7
Limestone, ground	1.0
Salt	0.4
L-Lys.HCl	0.1
Trace-mineral premix^a^	0.5
Vitamin premix^b^	0.5
Calculated chemical composition	
DE, MJ/kg	13.40
CP, %	16.00
Ca, %	0.62
P, total, %	0.49
P, available, %	0.25
Lys, %	0.85
Methinone, %	0.24
Met + Cys, %	0.50
Thr, %	0.60
Trp, %	0.18

^a^supplied per kilogram of diet: 12 mg of Cu (sulfate), 150 mg of Zn (zinc oxide); 120 mg of Fe (iron sulfate monohydrate), 0.7 mg of I (calcium iodate); 45 mg of Mn (manganous oxide), and 0.3 mg of Se (sodium selenite)

^b^supplied per kilogram of diet: 4,950 IU of vitamin A (acetate), 660 IU of vitamin D_3_, 4.8 mg of vitamin K (menadione sodium bisulfate complex), 2.36 mg of Vitamin E, 16.5 mg of D-pantothenic acid, 6.0 mg of riboflavin, 33.0 mg of niacin, 24.5 μg of vitamin B_12_, and 3.3 mg of vitamin B_6_

Pigs were weighed after fasting for 12 h at the beginning and end of the 64-d finishing period to determine average daily gain (ADG). Feed and water were provided *ad libitum* throughout the entire experiment period. Average daily feed intake (ADFI) for pigs was calculated as feed offered minus feed refused every 7 d. Average daily gain: feed intake (G:F) was obtained based on ADG and ADFI. On d 64, heparinized blood (10 mL) was collected by jugular venipuncture from 1 gilt and 1 barrow in each pen, 4 h after feeding (1400 h). Blood was held on ice until centrifugation (3,000 × *g* for 15 min at 4 °C), aliquots of plasma were stored at -20 °C for subsequent analysis. The blood-sampled barrows (*n* = 6 in each treatment group) were then fasted for 12 h, with water available, weighed on d 65 and electrically stunned and exsanguinated. Back fat, abdominal fat, liver and longissimus muscle (6/7^th^ lumbar vertebra level) were immediately sampled, snap-frozen in liquid nitrogen, and stored at -80 °C for subsequent analysis. Back fat thicknesses at the first rib, 6/7^th^, 10^th^, last rib, and the last lumbar vertebra were measured as was longissimus muscle area between the 10^th^ and 11^th^ ribs. Eye muscle areas were measured from digital images of a slice of longissimus muscle taken between the 10^th^ and 11^th^ ribs.

### Meat quality traits

The following meat quality measurements were made on longissimus muscle.

#### Loin color components and pH

Loin color components and pH were assayed following the method of Cherel et al. [10]. The CIE L* (lightness), a* (redness), and b* (yellowness) values were determined from a mean of four random readings (two readings for each chop) at 45 min or 24 h postmortem using a Minolta chromameter CR-300 (Osaka, Japan), with a D65 illuminant and a 1-cm diameter aperture. The pH at 45 min, 24 h or 48 h postmortem was measured directly in longissimus muscle (7/8^th^ rib) using pH meter (Ingold Xerolyte electrode, Knick pH-meter, Berlin, Germany).

#### Drip loss, marbling score and shear force

After slaughter, two 2.5 cm-thick longissimus muscle chops (10/11^th^ rib) were visually evaluated for marbling (1 = devoid to 10 = abundant). The same day, one slices of longissimus muscle (approximately 100 g, 9/10^th^ rib to the last rib) were collected, trimmed of external fat and perimysium, weighed, and kept at 4 °C in a plastic bag for a subsequent 45 min, 24 h or 48 h for determination of drip loss after muscle were sampled [11]. Drip loss was calculated as a percentage: [(initial weight-final weight)/initial weight] × 100. According to Trefan et al. [12], shear force value (expressed in Newtons) was measured perpendicular to the axis of muscle fibers in 8 replicates for each sample.

#### Intramuscular fat determination

Muscle slices were also taken on the last rib longissimus muscle, trimmed of external fat, minced, and freeze-dried before determination of intramuscular fat content after chloroform-methanol extraction, as described previously [13]. Lipid content of fresh tissue (g/100 g) was calculated by taking into account the dry matter content determined from the weight of minced tissue before and after freeze-drying.

### Antioxidant enzyme activity

Activities of catalase (CAT), glutathione peroxidase (GPx), and total superoxide dismutase (T-SOD), total antioxidant capacity (T-AOC), glutathione-S-transferase (GST) and γ-glutamylcysteine synthetase (γ-GCS) in plasma or muscle homogenates were measured in duplicate using commercial assay kits (Nanjing Jiancheng Bioengineering Institute, Nanjing, China) and a plate reader. The oxidized glutathione (GSSG) and reduced glutathione (GSH) concentration in plasma were assayed according to the kit instruction (Nanjing Jiancheng Bioengineering Institute, Nanjing, China). Supernatants, after perchloric acid extraction (muscle and liver homogenized in 4 vol 1 mol/L cold acid and centrifugation) were used to measure GSSG and reduced GSH content with a kit from the same company. Enzyme activity, GSSG and reduced GSH content in muscle and liver were standardized against protein concentrations.

### Plasma prooxidant-antioxidant balance (PAB) assay

A PAB method, slightly modified from that described by Alamdari et al. [14] was used for assay of plasma. Acetate buffer (50 mmol/L, pH 4.5) was used instead of phosphate:citrate buffer and pure 3,3′,5,5′-tetramethylbenzidine (TMB) in dimethyl sulfoxide (DMSO) was used instead of reagent tablets. The assay “working solution” was essentially the same and conditions for performing the assay and expressing PAB were almost identical. Full details of the modifications are available upon request. The values of the PAB are expressed in arbitrary HK units, being the percentage of hydrogen peroxide in the standard solution.

### Determination of tissue MDA content

The extent of lipid oxidation in plasma, liver, longissimus muscle, backfat and abdominal fat was determined by measuring levels of malondialdehyde (MDA), a secondary lipid oxidation product. The thiobarbituric acid method of Raharjo et al. [15] was used and results were expressed as nmol/L for plasma and nmol/mg protein in solid tissues; protein was measured by the BCA method.

### Isolation of RNA and real-time PCR

Total RNA was extracted from muscle, liver, backfat and abdominal fat using TRIzol reagent (Invitrogen, Carlsbad, CA, USA) according to the manufacturer’s instructions. All RNA samples were treated with DNAase (TAKARA, Dalian, China) and were of high quality as determined by OD_260:280_ and evaluation after gel electrophoresis. Complementary DNA was prepared by reverse-transcription using TAKARA RT reagents according to the manufacturer’s instructions. Real-time PCR was performed on 1 μL of cDNA product in a total volume of 20 μL containing 10 μL of SYBR-green PCR master Mix (TAKARA, Dalian, China) and 0.2 μmol/L of gene-specific forward and reverse primers (Table 2). The following protocol was used: denature at 95 °C for 30 s, followed by 40 cycles of 95 °C for 20 s, 60 °C for 30 s, and 72 °C for 20s. The relative quantification of target gene expression was evaluated by normalizing its signal to that of β-actin using 2^-ΔΔCt^ method [16]. The fold difference in the relative gene expression of target was calculated as the 2^-ΔΔCt^ value.

**Table 2:** Oligonucleotide polymerase chain reaction primers

Gene	Primer sense/antisense	Product length, bp
*β-actin*	CCAGGTCATCACCATCGG CCGTGTTGGCGTAGAGGT	158
*COX1*	TGGCAACTGCTTCTTCCCTTT GTGAGCCGACTGAACACCATCTAT	163
*COX2*	AGCAGGCTGATACTGATAGGAG TGTTGATAGTTGTACTCGTGGC	224
*GR*	GTGAGCCGACTGAACACCATCTAT CTTCTTCCCGTTGACCTCTACTG	120
*GCL*	AACCAGGCTCTCTGCACAATCACTT TTAGGGTACTGAAACGCGGGTGC	226
*NOX2*	ACCCTTTCACCCTGACCTCT AATCCCTGCTCCCACTAACA	221
*NOX4*	TGGAACGCACTACCAGGATG TTCGGCACAATACAGGCACA	202
*NOX5*	GCCTGGCGACTACTTGTATCTG CTTCCTCTGACTCCTTCTCATTTTC	226
*5-LOX*	GCCAGTGGTTTGCGGGCA GCTTCTCGATTTTGATGAGCTGGA	183
*P450 8B1*	AAGGATGCGAAGAGAAAACTAGACT	122
	AGGTGCTTGGTGCTGGCTGA	

### Statistical analysis

The results are presented as the mean ± SE. Body weight (initial and ending), ADFI, ADG and G:F were analyzed using one-way ANOVA. Following the method of White et al. [17], the statistical model included dietary supplementation, replicate, and the interaction of dietary supplementation × replicate as sources of variation. Means were compared using preplanned pairwise *t*-test. Calculations were made using PROC MIXED and PDIFF option (SAS Inst. Inc., Cary, NC). Drip loss, pH and color of meat were analyzed using one-way ANOVA with repeated measures. The statistical model included dietary supplementation, replicate, time and all two- and three-way interactions as sources of variation. Pig with dietary supplementation × replicate was used as random variable in the model. Means were compared using a preplanned pairwise *t*-test. Calculations were made using PROC MIXED of SAS with the REPEATED statement. The back fat was analyzed using one-way ANOVA with repeated measures. The statistical model included dietary supplementation, replicate, backfat location and all two- and three-way interactions as sources of variation. Means were compared using preplanned pairwise *t*-tests. Calculations were made using PROC MIXED of SAS, and means were separated using PDIFF option of SAS.

## Results

**Fig. 1:**
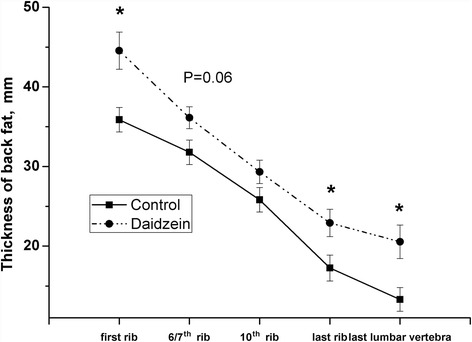
Effects of high-level supplementation with daidzein on the thickness of backfat in finishing barrows. “*” indicates significantly different from control (*P* < 0.05). Values are means with bars represent “SE ”, *n* = 6

As shown in Table 3, the ADFI in pigs fed daidzein was 7.5 % greater than control pigs (*P* < 0.05). There were no significant differences between the two groups in average daily gain (ADG) and G:F (Table 3). Compared to the control animals, pigs consuming daidzein had higher back fat thickness (*P* < 0.05) over the first and last ribs, and the last lumbar vertebra (Fig. 1).

**Table 3:** Effects of high-level supplementation with daidzein on growth performance in finishing pigs^a^

Variables	Control	Daidzein	SEM	*P*-value
Initial body weight, kg	57.45	57.47	1.06	0.99
Final body weight, kg	110	112	2.27	0.65
Average weight gain, g/d	914	940	26.4	0.50
Average feed intake, g/d	2,871	3,086	53.8	0.02
G:F^b^	0.32	0.30	0.01	0.15

^a^Values are means (*n* = 6)

^b^G:F, average weight gain:feed intake

The antioxidant indices in plasma are summarized in Table 4. Compared with control, supplementation of daidzein resulted in higher activity of plasma γ-GCS in barrows but not in gilts. The plasma activities of SOD, GST and CAT were not affected either in barrows or in gilts. The plasma PAB value in barrows fed supplemental daidzein was approximately 35 % greater than in the controls (*P* < 0.05), indicating pro-oxidant potential of daidzein in the circulation.

**Table 4:** Changes in plasma antioxidant enzymes of finishing pigs fed a high dose of supplemental daidzein^a^

Variables	Control	Daidzein	SEM	*P*-value
γ-GCS^b^, U/mL				
Barrow	3.46	5.23	0.49	0.05
Gilt	3.56	5.20	0.74	0.15
T-SOD^c^, U/mL				
Barrow	53	58.48	2.66	0.18
Gilt	54.11	53.94	3.12	0.16
T-AOC^d^, U/mL				
Barrow	1.49	0.98	0.23	0.16
Gilt	1.24	1.36	0.27	0.70
GST^e^, U/mL				
Barrow	21.10	18.31	1.6	0.25
Gilt	22.38	19.27	1.28	0.12
CAT^f^, U/mL				
Barrow	4.73	4.15	0.86	0.66
Gilt	3.79	3.63	0.68	0.86
GSH/GSSG^g^				
Barrow	1.75	1.78	0.66	0.95
Gilt	1.57	1.69	0.39	0.86
PAB value^h^				
Barrow	39.31	53.19	1.17	<0.01
Gilt	45.02	42.96	0.74	0.23

^a^Values are means (*n* = 6)

^b^γ-GCS, γ-glutamylcysteine synthetase activity

^c^T-SOD, total superoxide dismutase activity

^d^T-AOC, total antioxidant capacity

^e^GST, glutathione-S-transferase activity

^f^CAT, catalase activity

^g^GSH/GSSG, reduced glutathione/oxidized glutathione

^h^PAB, prooxidant-antioxidant balance

**Fig. 2:**
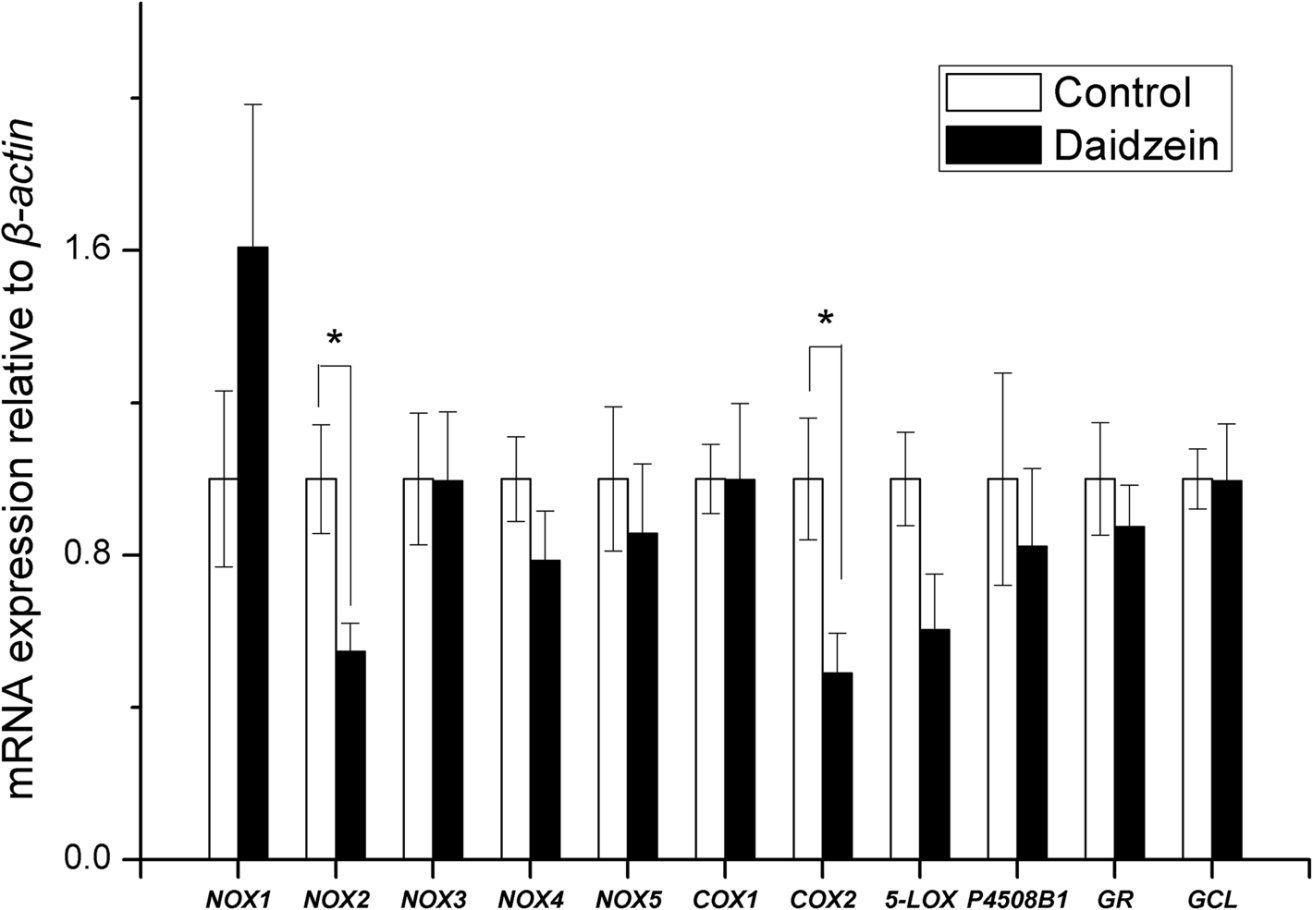
Effects of high-level supplementation with daidzein on relative transcript abundance of anti/pro-oxidant enzyme genes in muscle of finishing barrows. “*” indicates different from control (*P* < 0.05). Values are means, bars represent “SE”, *n* = 6. *NOX*, NADPH oxidase; *COX*, cyclooxygenase; *5-LOX*, 5-lipoxygenase; *GR*, glutathione reductase; *GCL*, glutamate cysteine ligase; *P4508B1*, cytochrome P-450 8B1

As shown in Table 5, pigs fed daidzein had higher muscle SOD activity and T-AOC than those of control animals (*P* < 0.05). The mRNA abundances of *NADPH oxidase-2* (*NOX2*) and *cooxygenase-2* (*COX2)* in longissimus muscle were significantly reduced in daidzein-fed pigs but there was no effect on expression of *glutathione reductase* (*GR*), *glutamate cysteine ligase* (*GCL*), *NOX4*, *NOX5*, *COX1*, *5-lipoxygenase* (*5-LOX*), or cytochrome *P450 8B1* (Fig. 2). There were no differences between controls and daidzein-fed barrows in marbling score, eye muscle areas, intramuscular fat content, shear force, drip loss, pH and color (Table 6), suggesting a negligible daidzein effect in finishing pigs on these indices of meat quality. Compared with control, pigs fed daidzein had higher CAT and SOD activity but had lower GSH/GSSG in liver (*P* < 0.05, Table 7).

**Table 5:** Changes in antioxidant indicators in the longissimus muscle of finishing barrows fed a high dose of supplemental daidzein^a^

Variables	Control	Daidzein	SEM	*P*-value
GSH/GSSG^b^	0.066	0.062	0.004	0.45
GPx^c^, U/mg pro	1.16	1.13	0.27	0.94
T-AOC^d^, U/mg pro	0.036	0.063	0.01	0.05
T-SOD^e^, U/mg pro	14.71	17.56	0.81	0.02

^a^Values are means (*n* = 6)

^b^GSH/GSSG, reduced glutathione/oxidized glutathione

^c^GPx, glutathione peroxidase activity

^d^T-AOC, total antioxidant capacity

^e^T-SOD, total superoxide dismutase activity

**Table 6:** Effects of high-level supplementation with daidzein on indices of meat quality in finishing pigs^a^

Variables	Control	Daidzein	SEM	*P*-value
Marbling score^b^	3.25	3.54	0.21	0.36
Eye muscle areas, cm^2^	49.97	48.92	3.85	0.85
Intramuscular fat, %	2.23	1.94	0.36	0.57
Shear force, Newton	51.11	51.07	2.43	0.99
Drip loss, %				
24 h	1.68	1.69	0.06	0.89
48 h	2.47	2.35	0.11	0.47
pH				
45 min	6.31	6.35	0.10	0.76
24 h	5.53	5.50	0.03	0.59
48 h	5.54	5.48	0.07	0.55
Color				
45 min				
L*^c^	44.13	44.15	1.21	0.99
a*	19.00	19.23	2.23	0.94
b*	3.41	3.20	0.62	0.82
24 h				
L*	53.48	54.86	1.13	0.41
a*	17.90	15.93	1.58	0.40
b*	3.59	3.79	0.58	0.82
48 h				
L*	54.54	54.90	1.22	0.84
a*	16.11	15.78	0.51	0.69
b*	3.46	3.19	0.44	0.70

^a^Values are means (*n* = 6)

^b^Marbling scores: 1 = devoid to 10 = moderately abundant or greater

^c^The L* variable represents lightness, 0 for black and 100 for white; a* represents the intensity in red; and b represents the intensity in yellow

**Table 7:** Changes in antioxidant indicators in the liver of finishing barrows fed a high dose of supplemental daidzein^a^

Variables	Control	Daidzein	SEM	*P*-value
GSH/GSSG^b^	0.16	0.07	0.03	0.03
CAT^c^, U/mg pro	79.5	96.3	2.27	<0.01
T-AOC^d^, U/mg pro	0.64	0.70	0.05	0.44
T-SOD^e^, U/mg pro	285	358	16.7	0.01

^a^Values are means (*n* = 6)

^b^GSH/GSSG, reduced glutathione/oxidized glutathione

^c^CAT, catalase activity

^d^T-AOC, total antioxidant capacity

^e^T-SOD, total superoxide dismutase activity

**Fig. 3:**
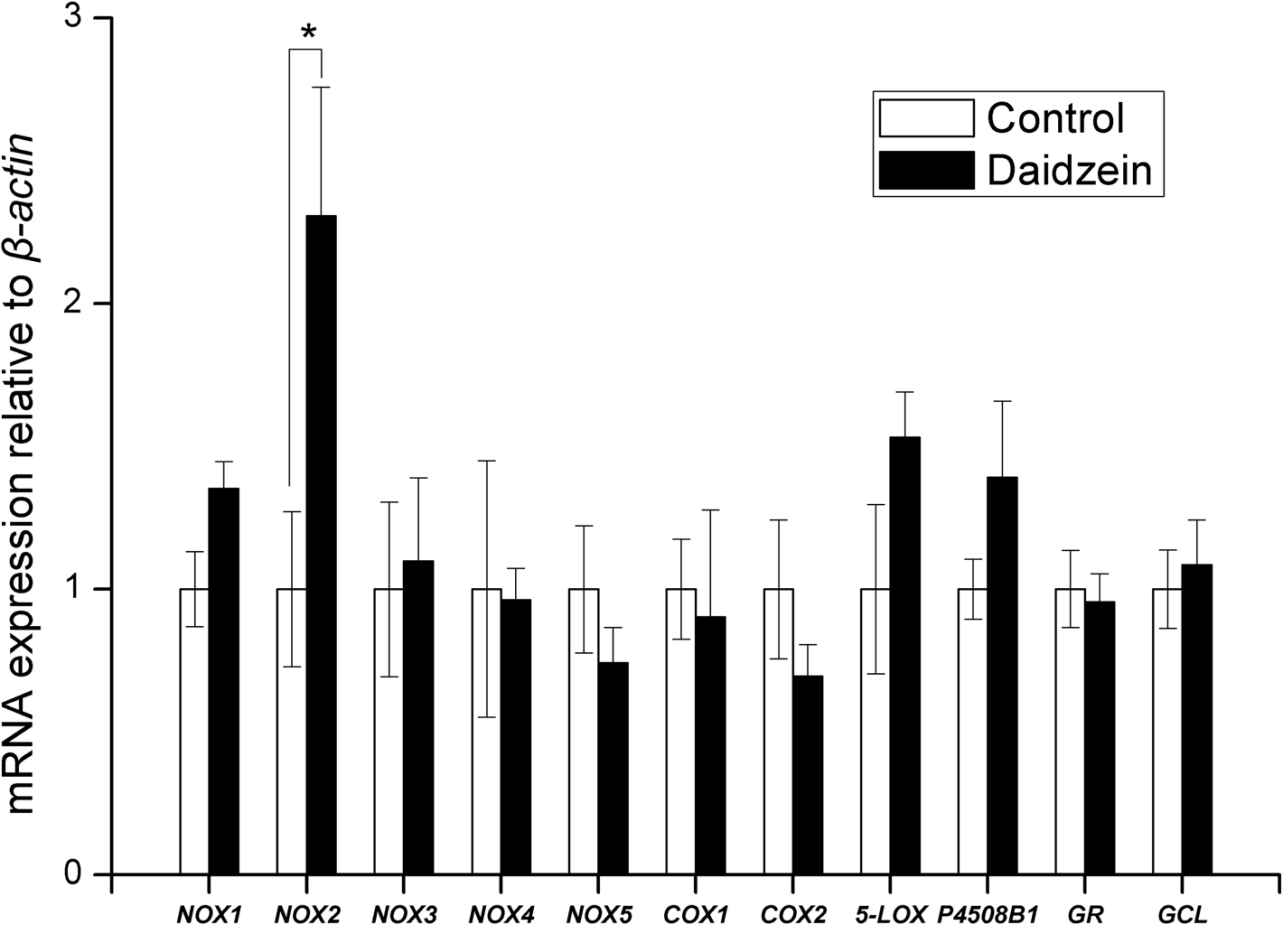
Effects of high-level supplementation with daidzein on relative transcript abundance of anti/pro-oxidant enzyme genes in liver of finishing barrows. “*” indicates different from control (*P* <0.05). Values are means, bars represent “SE”, *n* = 6. *NOX*, NADPH oxidase; *COX*, cyclooxygenase; *5-LOX*, 5-lipoxygenase; *GR*, glutathione reductase;* GCL*, glutamate cysteine ligase; *P4508B1*, cytochrome P-450 8B1

**Fig. 4:**
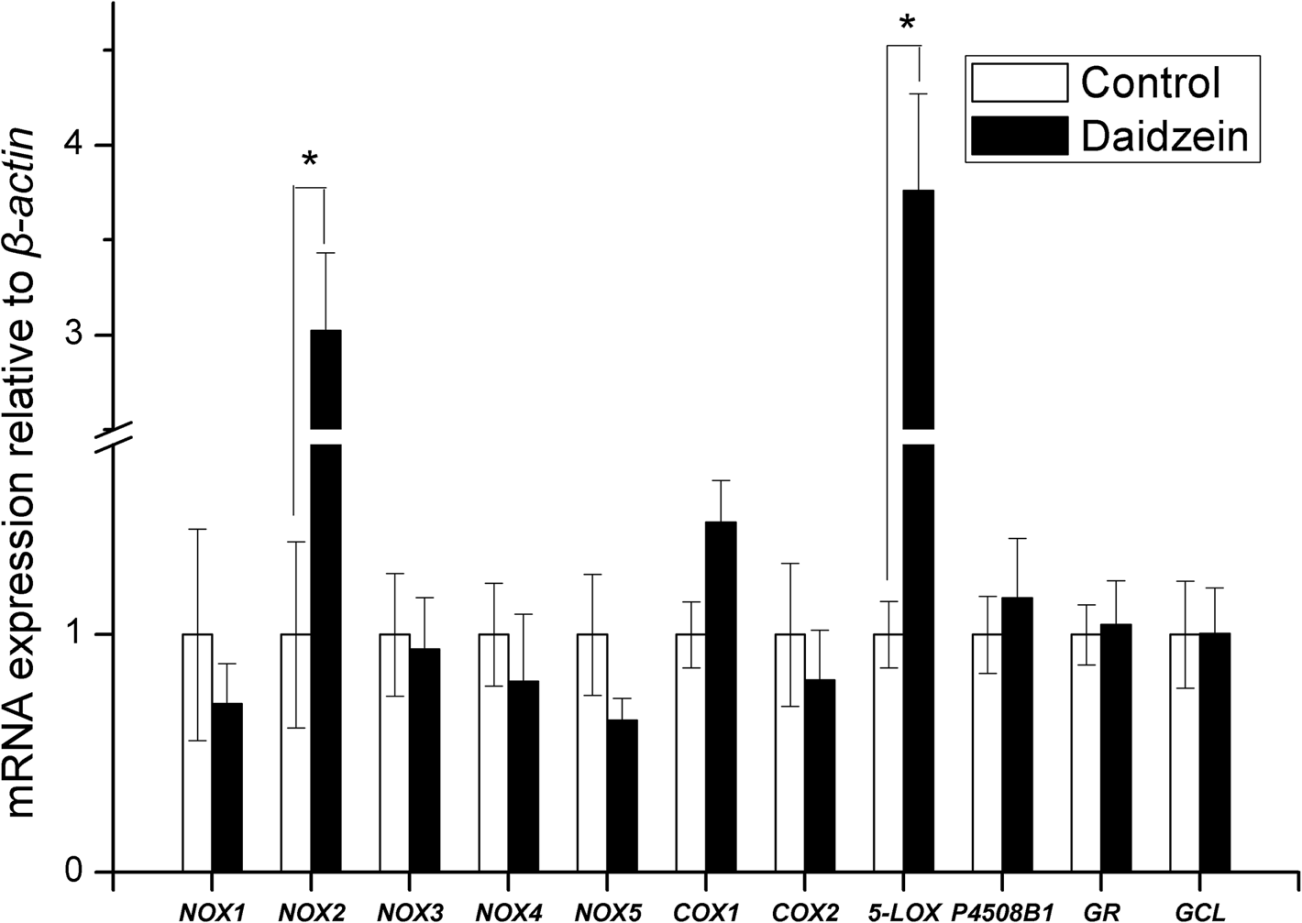
Effects of high-level supplementation with daidzein on relative transcript abundance of anti/pro-oxidant enzyme genes in backfat of finishing barrows. “*” indicates different from control (*P* <0.05). Values are means, bars represent “SE”, *n* = 6. *NOX*, NADPH oxidase; *COX*, cyclooxygenase; *5-LOX*, 5-lipoxygenase; *GR*, glutathione reductase; *GCL*, glutamate cysteine ligase; *P4508B1*, cytochrome P-450 8B1

**Fig. 5:**
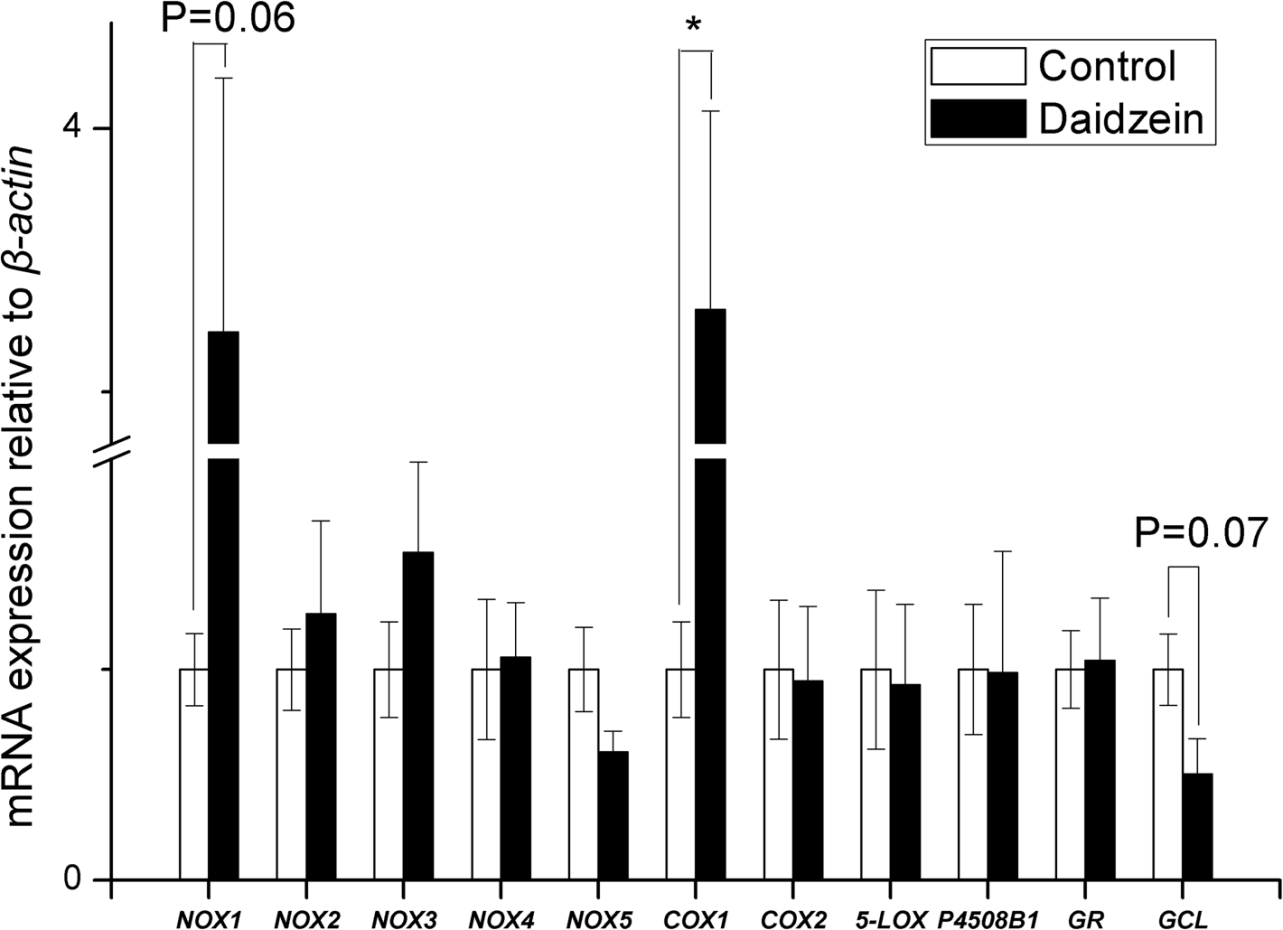
Effects of high-level supplementation with daidzein on relative transcript abundance of anti/pro-oxidant enzyme genes in abdominal fat of finishing barrows. “*” indicates different from control (*P* <0.05). Values are means, bars represent “SE”, *n* = 6. *NOX*, NADPH oxidase; *COX*, cyclooxygenase; *5-LOX*, 5-lipoxygenase; *GR*, glutathione reductase; *GCL*, glutamate cysteine ligase; *P4508B1*, cytochrome P-450 8B1

*NOX2* mRNA expression in abdominal fat was 130 % higher in daidzein-fed barrows than control (*P* < 0.05), while there were no differences observed in *NOX1*, *NOX4*, *COX1*, *COX2*, *NOX5*, *5-LOX* and *P450 8B1* (Fig. 3). The transcript abundance of both *NOX2* and *5-LOX* in the backfat of daidzein-fed pigs was almost 2 to 3 times higher than that of control (*P* < 0.05, Fig. 4). In liver, the gene mRNA abundance of *COX1* were higher in daidzein-fed pigs than control (*P* <0.05) but *NOX1* expression tended to be higher in daidzein-fed pigs (*P* = 0.06, Fig. 5). Liver *GCL* mRNA abundance tended to be higher in daidzein-fed pigs than control (*P* = 0.07, Fig. 5).

**Fig. 6:**
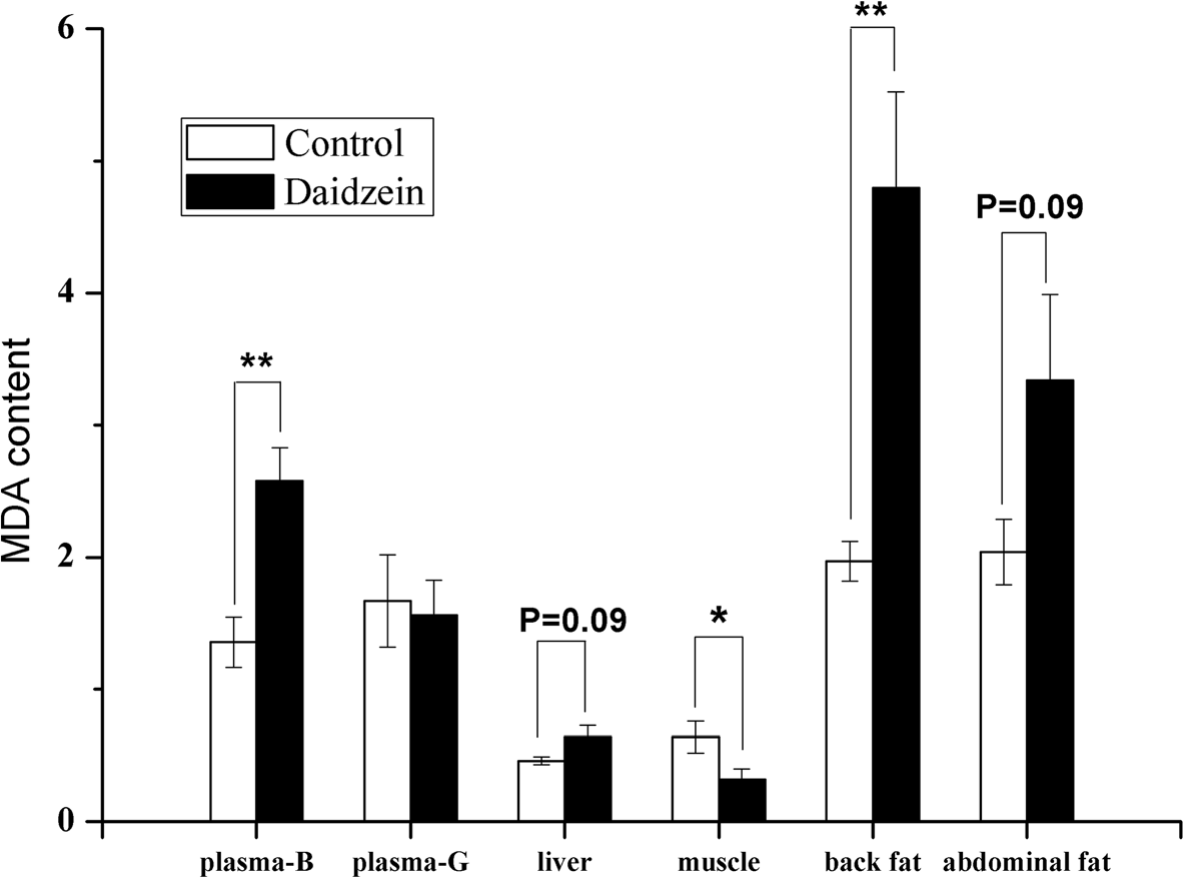
The effects of high-level dietary supplementation with daidzein on the malondialdehyde (MDA) content in various tissues of finishing pigs. MDA contents are expressed as nmol/mg protein in solid tissues and as nmol/L in plasma (B, barrows; G, gilts). Values are means (*n* = 6), bars represent “SE”. “*”, “**” indicates significantly different from control (*P <0.05, **P <0.01)

Plasma concentrations of MDA in daidzein-fed barrows, but not gilts, were 190 % those of controls (*P* <0.01, Fig. 6). Compared with control, the daidzein-fed barrows had higher MDA content in liver, abdominal fat (*P* = 0.09) and back fat (*P* <0.01) but had lower MDA content in longissimus muscle (*P* = 0.05).

## Discussion

In the present study, consumption of a high level of dietary daidzein increased average feed intake, but the increase did not lead to greater daily gain. Kishida et al. [18] found that dietary supplementation with compound of daidzein and genistein resulted in reduced feed intake in female rats but not in male rats. The present study has shown increased thickness of thoracic and lumbar back fat (first and the last rib and last lumbar vertebra), suggesting a stimulatory effect of daidzein on fat deposition in these finishing barrows. In 3 T3-L1 cells, daidzein enhanced adipocyte differentiation and PPARγ expression in a dose-dependent manner [19], and supplementation of 50 mg/kg dietary genistein increased the weight of epididymal and renal fat pads in male mice [20]. Other studies show quite different outcomes. Ovariectomized adult female mice, supplemented with 1500 mg/kg dietary genistein for 3 wk had reduced fat pads and body weight, and increased apoptosis in adipose tissue [21]. Daidzein (50 mg/kg BW, i.p.) reduced short-term feed intake of rats and down-regulated the fatty acid synthesis-related gene expression in adipose tissue [22]. Daidzein was also shown to inhibit the adipogenesis in mesenchymal stem cells through stimulation of lipolysis [23]. Additionally, administration of 450 mg/(kg×d) soy isoflavones caused reduction in the body weight and deposition of visceral adipose tissue in high-fat-diet induced insulin resistant rats [24].

Reactive oxygen species (ROS) are normal metabolic products and play important roles in mediating cell function, including cell signaling [25] and protection against environmental insults, both biological and chemical [25, 26]. Imbalance between ROS production and scavenging systems results in oxidative injury to proteins, DNA, and lipids [27]. Several enzyme systems contribute to the production of ROS, including NOX, xanthine oxidase, COX and P450 [28] while SOD, GPx and CAT are the principle antioxidant enzymes that eliminate cellular ROS, and GSH provides non-enzymatic defense [29]. These anti-and pro-oxidant systems were evaluated here to assess the pro-oxidant potential of daidzein, at high level supplementation. The down-regulation of NOX2 and COX2 in longissimus muscle of finishing pigs was unexpected and suggests a suppressive effect of the high dose of daidzein on the pro-oxidant system. Until now, there was no direct evidence of daidzein influencing the NOX system though similar dietary supplementation with genistein (500 mg/kg) and equol (250 mg/kg) conferred neuroprotection in rats by reducing NOX activity and upregulating antioxidant genes [30]. Similarly, a moderate concentration of genistein (50 or 100 μmol/L) suppressed expression of the p22phox NADPH oxidase subunit in aortic endothelial cells from stroke-prone spontaneously hypertensive rats [31]. Consistent with the present results, the inhibitory role of genistein in regulating NADPH oxidase was also demonstrated in human oral squamous carcinoma cells [32] and in a diabetic mouse model subjected to chronic i.p. treatment with genistein [33].

Another ROS-producing system in oxidative stress, COX, is involved in prostaglandin synthesis [34] and *COX2* expression in muscle of the finishing pigs was down-regulated by high-dose supplementation with daidzein. A major metabolite of daidzein, 7,3′,4′-trihydroxyisoflavone (THIF), inhibited ultraviolet B-induced COX2 expression through inhibition of nuclear factor (NF-κB) transcriptional activity in mouse epidermal JB6 P+ cells [35]. The enhanced ROS-scavenging enzyme (SOD), as well as the suppressed ROS-inducing enzyme (NADPH oxidase and cyclooxygenase) in the muscle of pigs fed high dose of daidzein, represents the shift towards antioxidant in the pro/antioxidant balance, which finally contributes to the reduced lipid peroxidation in muscle. The present results indicate that high-dose dietary daidzein increased antioxidant enzymes in muscle so both processes contribute to an improved redox status. Despite this outcome, there was no overall effect of daidzein on the meat quality.

In contrast to the effects in muscle, daidzein appeared to exert pro-oxidant potential in liver, back fat and abdominal fat, based on levels of the lipid peroxidation marker MDA in these tissues. There were significant increases in the plasma concentrations of MDA and PAB value in just the barrows, indicating a pro-oxidant effect of the high-level supplementation with daidzein. The increased plasma activity of γ-GCS is probably a feed-back response to pro-oxidative effects of daidzein. The expression of *NOX2* was up-regulated by daidzein both in backfat and abdominal fat, which is contrast to that observed in muscle. Similarly, significant up-regulation of *COX1* and* NOX1* in liver as well as increased expression of *5-LOX* in backfat were also observed in the pigs fed high dose of daidzein. In accordance with the results, the recent report showed that high concentration of genistein, with similar chemical structure to daidzein, increased cellular ROS production by up-regulating 5-LOX [36]. Likewise, it has been reported that LOX mediates the pro-oxidative effect of the anti-oxidant melatonin via stimulation of arachidonic acid metabolism [37]. Lipoxygenases (LOX), an iron-containing dioxygenase, can metabolize arachidonic acid to generate a variety of bioactive eicosanoids, including prostaglandins and leukotrienes [38]. During the catalytic cycle of LOX, peroxyl radical complexes are formed and they can serve as sources of free radicals [39]. The ability of daidzein at high dose to elicit the activation of pro-oxidant enzyme system provides a possible mechanism to explain why lipid peroxidation occurred in fat and liver when fed high dose of daidzein. Still it could not be excluded that the pro-oxidant function may be mediated through its metabolism because it is reported that 7,3′,4′-trihydroxyisoflavone (7,3′,4′-THIF), one of the major metabolites of daidzein was able to increase the ROS production in human cervical cancer cells [40]. The results of this study indicate that daidzein supplementation led to pro- or anti-oxidant effects in a tissue-dependent manner.

The biological actions of isoflavones vary, depending upon their concentrations. Low concentrations enhance the antioxidant system [3, 4] and protect cells against oxidative stress [41, 42] while high concentrations may cause oxidative injury, such as DNA damage and cell death [36, 43]. The basis for the different effects of daidzein in muscle from other tissues, shown here, is not known but might reflect differential daidzein uptake or sensitivity in the various tissues, possibly related to its lipid solubility.

## Conclusion

In summary, this study has demonstrated that high-level supplementation of a corn-soybean meal diet with daidzein enhances the redox system in the longissimus muscle of finishing pigs by down-regulating the pro-oxidant system and is without effect on indices of meat quality. At the same time, pro-oxidant responses were apparent in liver and fat tissue, suggesting that tissue-dependent actions existed.

## Abbreviations

5-LOX, 5-lipoxygenase; CAT, catalase; COX, cyclooxygenase; DMSO, dimethyl sulfoxide; GCL, glutamate cysteine ligase; GPx, glutathione peroxidase; GR, glutathione reductase; GSH, reduced glutathione; GSSG, oxidized glutathione; GST, glutathione-S-transferase; MDA, malondialdehyde; NOX, NADPH oxidase; P4508B1, cytochrome P-450 8B1; PAB, prooxidant-antioxidant balance; PPARα, peroxisome proliferator-activated receptor alpha; ROS, reactive oxygen species; T-AOC, total antioxidant capacity; TMB, 3,3′,5,5′-tetramethylbenzidine; T-SOD, total superoxide dismutase; γ-GCS, γ-glutamylcysteine synthetase.
